# 777 Food Swamps, Food Deserts, and Wound Healing in Burn Patients at an Urban Academic Center

**DOI:** 10.1093/jbcr/irad045.252

**Published:** 2023-05-15

**Authors:** Brienne Donovan, Daniel Wiese, Jingwei Wu, Lisa Rae, Jeffrey Anderson, Kevin Henry

**Affiliations:** Temple University, Philadelphia, Pennsylvania; Temple University, Philadelphia, Pennsylvania; Temple University, Philadelphia, Pennsylvania; Temple University, Philadelphia, Pennsylvania; Temple University, Philadelphia, Pennsylvania; Temple University, Philadelphia, Pennsylvania

## Abstract

**Introduction:**

Many victims of burn injury live in food deserts or food swamps. Food deserts are low-income areas with minimal or no access to nutritious foods while food swamps are areas with much greater access to unhealthy, high-calorie foods. We examined the relationship between residency in a food swamp or food desert by census tract and wound healing.

**Methods:**

We performed a retrospective review of burn patients who underwent split-thickness skin grafting at an ABA verified urban academic burn center between September 2018 and August 2022. Inclusion criteria were burn area less than 20% total body surface area (TBSA), age ≥ 18, and single operation for split-thickness skin grafting. Patient residential locations were used in conjunction with Modified Retail Food Environment Index (mREFI) data to classify residency in food deserts and food swamps. The primary outcome was time to donor site healing. Time-to-event Cox proportional regression analysis was performed to evaluate risk factors for poor wound healing.

**Results:**

A total of 238 patients were included in the study, of whom 110 (46.2%) were female and 128 (53.8%) were male. Mean age was 48, BMI was 28.5, TBSA 3.7, and average days to donor site healing was 26. In categorizing residency by mREFI data, 45 (18.9%) individuals had good access, 11 (4.6%) resided in food deserts, and 182 (76.5%) resided in food swamps. Although time to healing was longer on average in the food desert (25.8 days) and food swamp (26.4 days) groups compared to the group with good access to food within ½ mile (22.9 days), this difference was not statistically significant. Not having diabetes was found to be associated with shorter time to wound healing (HR=1.68, 95% CI [1.15, 2.47], p=0.007). Living in a good access area was associated with faster wound healing (compared to swamp: HR=1.23, 95% CI [0.86, 1.75], compare to desert: HR=1.06, 95% CI [0.51, 2.20]), however this association was not statistically significant.

**Conclusions:**

Residency in a food swamp or food desert does not significantly influence time to healing in burn injured patients. However, not having diabetes was associated with a shorter time to wound healing. This is the first study to compare residency in food deserts or food swamps by census tract level data with time to wound healing in an urban burn population.

**Applicability of Research to Practice:**

Wound healing is a complex and important process known to be affected by malnutrition. Our data contributes to the current body of literature on nutrition and wound healing.

